# Direction and magnitude of natural selection on body size differ among age‐classes of seaward‐migrating Pacific salmon

**DOI:** 10.1111/eva.12957

**Published:** 2020-04-09

**Authors:** Marta E. Ulaski, Heather Finkle, Peter A. H. Westley

**Affiliations:** ^1^ Department of Fisheries College of Fisheries and Ocean Sciences University of Alaska Fairbanks Fairbanks Alaska; ^2^ Alaska Department of Fish and Game Kodiak Alaska

**Keywords:** age‐class, body size, natural selection, ocean entry, *Oncorhynchus nerka*, selection differential, smolt, sockeye salmon

## Abstract

Due to the mediating role of body size in determining fitness, the “bigger‐is‐better” hypothesis still pervades evolutionary ecology despite evidence that natural selection on phenotypic traits varies in time and space. For Pacific salmon (genus *Oncorhynchus*), most individual studies quantify selection across a narrow range of sizes and ages; therefore, uncertainties remain concerning how selection on size may differ among diverse life histories. Here, we quantify the direction and magnitude of natural selection on body size among age‐classes of multiple marine cohorts of *O. nerka* (sockeye salmon). Across four cohorts of seaward migrants, we calculated standardized selection differentials by comparing observed size distributions of out‐migrating juvenile salmon to back‐calculated smolt length from the scales of surviving, returning adults. Results reveal the magnitude of selection on size was very strong (>90th percentile compared to a database of 3,759 linear selection differentials) and consistent among years. However, the direction of selection on size consistently varied among age‐classes. Selection was positive for fish migrating to sea after two years in freshwater (age 2) and in their first year of life (age 0), but negative for fish migrating after 1 year in freshwater (age 1). The absolute magnitude of selection was negatively correlated to mean ocean‐entry timing, which may underpin negative selection favoring small age‐1 fish, given associations between size and timing of seaward migration. Collectively, these results indicate that “bigger is not always better” in terms of survival and emphasize trade‐offs that may exist between fitness components for organisms with similarly diverse migratory life histories.

## 1 INTRODUCTION

Few phenotypic traits are as important as body size given its influence on fitness across taxa (Brown, Marquet, & Taper, 1993; Choudhury, Black, & Owen, 1996; Sokolovska, Rowe, & Johansson, 2000; Wikelski & Romero, 2003). In many species, traits associated with reproduction and survival are strongly influenced, both directly and indirectly, by body size. Body size is often positively correlated with female fecundity (Coates, 1988; Honek, 1993), can mediate the outcome of territorial aggression (Hastings, 1989; Johnsson, Nobbelin, & Bohlin, 1999; Tokarz, 1985), and affect the probability of starvation and rate of predation (Gliwicz, 1990; Scharf, Juanes, & Rountree, 2000; Sogard, 1997). Yet, despite the fitness benefits of increased body size, countervailing selection can occur as a result of the costs of increased detectability, higher energy requirements, and reduced agility for individuals of a larger size (Blanckenhorn, 2000). For example, hypoxia limits body size in *Drosophila melanogaster* that otherwise are strongly selected for larger mass (Klok & Harrison, 2009). Similarly, obtaining a large size before overwintering can be costly under certain conditions for juvenile *Oncorhynchus mykiss* (steelhead trout), where relatively warm winter temperatures and low food availability appeared to be more physiologically demanding for large fish (Connolly & Petersen, 2003). Furthermore, divergent selection can simultaneously favor both large and small body size in a population depending on the season and alternative strategies (Gross, 1985; Siepielski, Dibattista, & Carlson, 2009). These few examples, of many, demonstrate that the expression of body size reflects the balance between fitness costs and gains.

Ecological agents of selection that represent the causes of evolution (e.g., predation, parasitism) vary across the landscape and years (Bell, 2010; Calsbeek, Gosden, Kuchta, & Svensson, 2012; Carlson & Quinn, 2007; MacColl, 2011). Selection can also be opposing at different stages of the life history, resulting in trade‐offs between fitness components, which in turn can underpin life‐history divergence and adaptive radiations (Schluter, Price, & Rowe, 1991). Life‐history trade‐offs are predicted to be particularly strong in migratory or metamorphosing species, because individuals are exposed to selection across ecosystems within and among generations. Phenotypic traits that prove beneficial in one environment may incur a fitness disadvantage in the next or vice versa (Gillis, Green, Middleton, & Morrissey, 2008; Schluter et al., 1991; Waples, Teel, Myers, & Marshall, 2004). Furthermore, body size is often associated with the phenology of key life‐history events, such as migratory timing, that are predicted to occur when conditions are most favorable (Cushing, 1990).

Pacific salmon and other migratory *Oncorhynchus* spp. are ideal species for exploring fitness trade‐offs as a result of selection on traits, such as body size and migratory timing (Quinn, Doctor, Kendall, & Rich, 2009). For anadromous *Oncorhynchus* spp., high rates of mortality occur during a brief period after juveniles (i.e., “smolts”) enter the ocean (Healey, 1982; Parker, 1971), and correspondingly, population dynamics are largely influenced by this life stage (Cunningham, Westley, & Adkinson, 2018; Rogers & Schindler, 2011). Smolt size and ocean‐entry timing are two correlated traits that influence survival; however, with few exceptions, the ecological agents of selection (sensu MacColl, 2011) are usually unknown. In general, smolt‐to‐adult survival increases with increasing smolt size at time of ocean entry for anadromous salmonids (Foerster, 1954; Koenings & Geiger, 1993; Ward, Slaney, Facchin, & Land, 1989). Higher survivability of larger juvenile fish is thought to arise from increased escape ability, faster growth, and a shorter time to attain a size less susceptible to predation and starvation (Heintz & Vollenweider, 2010; Sogard, 1997).

Despite its intuitive appeal and general assumption in the literature, the evidence for size‐biased survival is equivocal. Size‐based patterns of survival are less obvious when comparing across populations spanning many degrees of latitude (Koenings & Geiger, 1993) or different brood years within populations (Henderson & Cass, 1991; Quinn, Dickerson, & Vøllestad, 2005). Positive size‐selective survival for freshwater age‐1 and age‐2 *O. kisutch* (coho salmon) smolts was detected only in years of poor marine conditions, suggesting that variability in ocean conditions may affect smolt growth rates and the susceptibility of smolts to a size‐selective predator (Holtby, Andersen, & Kadowaki, 1990). In addition, size at seaward migration for some species of Pacific salmon may not be as important as the size attained during their first marine growing season (Duffy & Beauchamp, 2011; Moss et al., 2005; Tomaro, Teel, Peterson, & Miller, 2012). For *O. kisutch,* the effects of smolt age at ocean entry can also affect size‐dependent survival because of the influence of out‐migration timing (Bilton, Alderdice, & Schnute, 1982). Similarly, earlier migrating age‐2 *O. nerka* smolts had lower marine survival than comparably sized age‐1 smolts, where age‐2 smolts needed to be approximately 20 mm larger to survive at a rate equal to age‐1 smolts (Koenings & Geiger, 1993).

Though the mechanisms of differential smolt survival across entry dates are generally unknown, smolt ocean entry relative to the spring bloom of their marine zooplankton prey is a predictor of within‐year variation in survival rates of *Oncorhynchus* spp*.* (Satterthwaite et al., 2014; Scheuerell, Zabel, & Sandford, 2009). For *O. tshawytscha* (Chinook salmon) juveniles entering the ocean from the Snake and Columbia rivers, an earlier migration was associated with increased survival although peak survival varied by ocean‐entry day among years (Scheuerell et al., 2009). Decreases in survival have been observed with a general increase in predatory fish such as the *Merluccius productus* (Pacific hake; Emmett, Krutzikowsky, & Bentley, 2006) and an increased predation rate on migrating juvenile salmon by colonial seabirds (Roby, Lyons, Craig, Collis, & Visser, 2003). Due to the variability in agents of selection over time, juvenile life‐history diversity provides stability and resilience to overall smolt production (Carr‐Harris et al., 2018; Hovel, Fresh, Schroder, Litt, & Quinn, 2018; Schroeder, Whitman, Cannon, Olmsted, & Rennie, 2016). This “bet‐hedging” strategy increases the probability that a component of smolts will experience favorable conditions and serve to buffer meta‐populations against environmental variability (Miller, Gray, & Merz, 2010; Moore, Mcclure, Rogers, & Schindler, 2010; Schindler et al., 2010). Though significant advancement has been made regarding our knowledge of the role of size‐selective survival for salmon smolts, most studies have been correlational by comparing mean smolt length across geographic regions, across years, and most often for a single age‐class (Henderson & Cass, 1991; Koenings & Geiger, 1993). Our understanding of how size‐selective pressure may affect contemporary evolution of size‐at‐age, migratory timing, and the maintenance of age structure within a single breeding population is more limited.

The purpose of this study was to understand the realized fitness advantages or disadvantages of body size across different life histories within a phenotypically diverse migratory population of *O. nerka*. The specific objectives of this study were to (a) quantify the magnitude and direction of natural selection on smolt size among age‐classes and cohorts, (b) compare observed selection on body size to a global database of selection on traits that includes 3,579 estimates from 91 species, and (c) determine the relationship of the magnitude of selection to mean ocean‐entry timing. We hypothesized that selection generally favors larger *O. nerka* smolts but that the magnitude of selection varies among age‐classes and among years. In addition, we hypothesized that the magnitude of selection will be positively correlated with late ocean‐entry timing.

## 2 MATERIALS AND METHODS

### 2.1 Model study system

**FIGURE 1:**
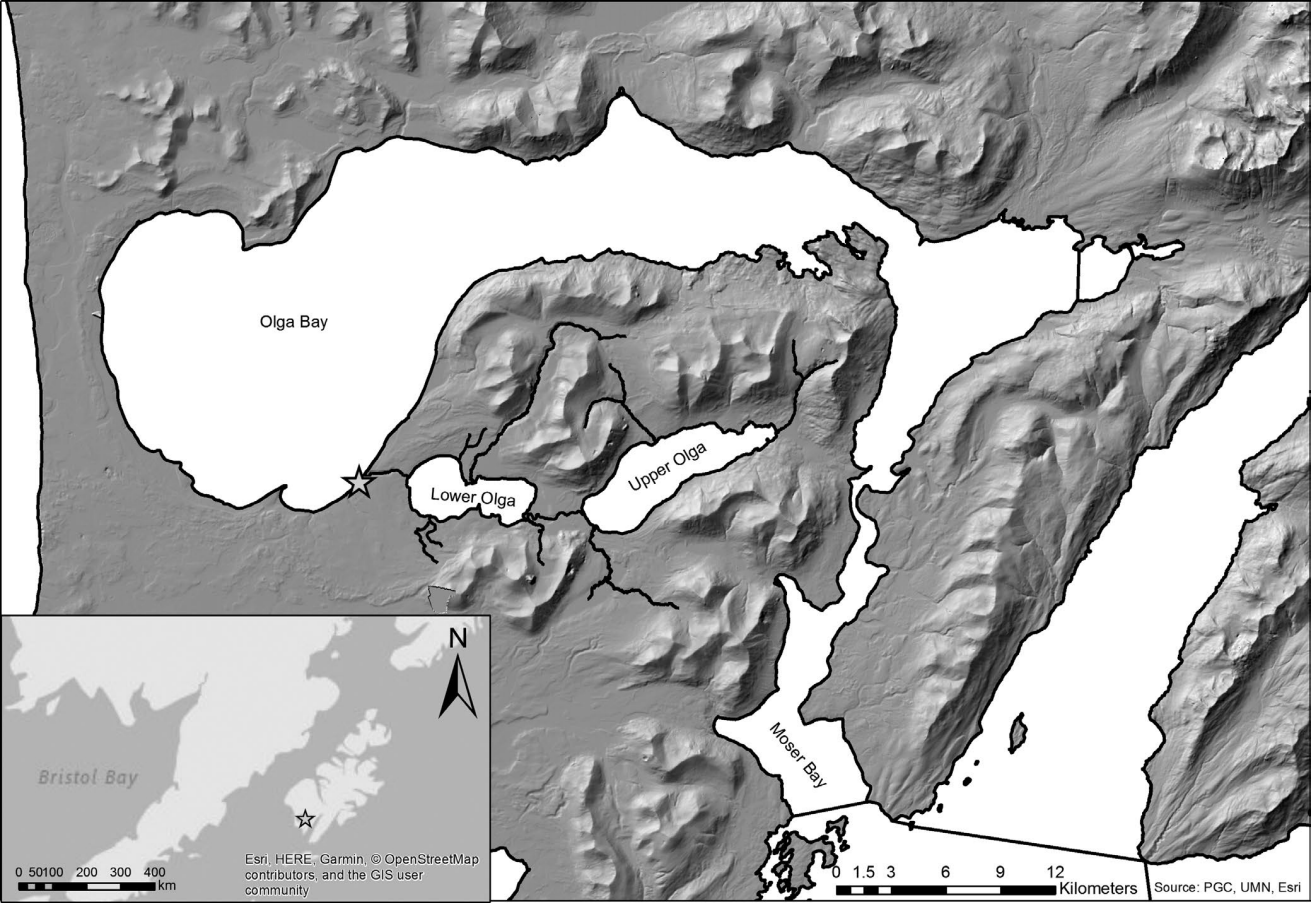
A map of Olga lakes, Kodiak, Alaska. The study area includes the Upper Station weir (star), Lower Olga Lake, and Upper Olga Lake

**FIGURE 2:**
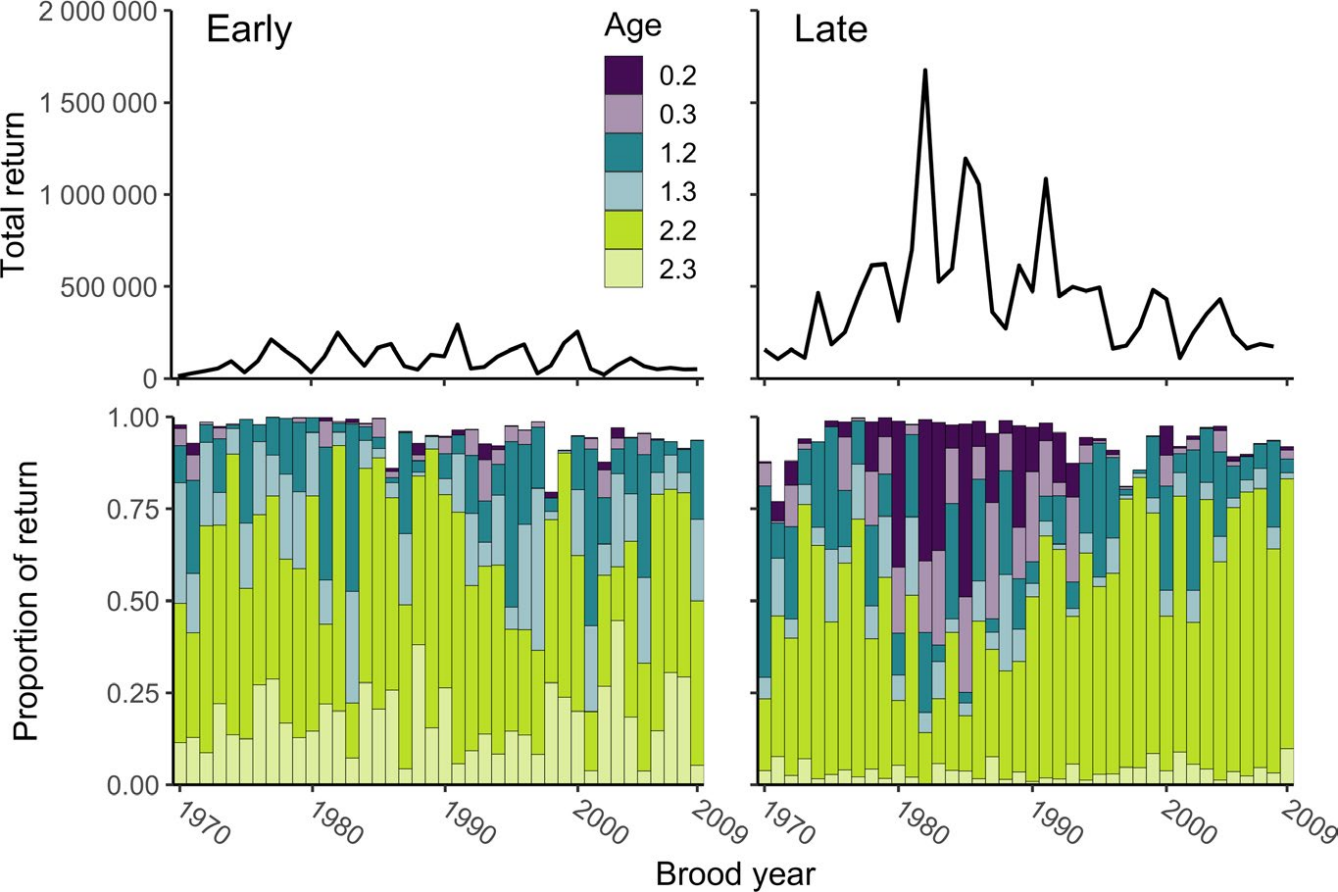
Estimated proportions of dominant age‐classes of Olga lakes Late Run *Oncorhynchus nerka* (bottom panel) out of total return by brood year (top panel). Ages are represented in European notation where the number before the decimal denotes years spent in freshwater and the number after the decimal indicates the number of years spent at sea

The South Olga lakes system on the southern end of Kodiak Island, Alaska, supports one of the largest *O. nerka* runs in the Kodiak Archipelago and has a long‐term average *O. nerka* run of approximately half a million fish (Finkle & Loewen, 2015; Jackson, Dinnocenzo, Spalinger, & Keyse, 2012). The system is composed of two lakes, Upper Olga Lake and Lower Olga Lake (Figure 1), and has two temporally distinct *O. nerka* runs that return from late May through mid‐July (Early Run) and from mid‐July through September (Late; Gomez‐Uchida, Seeb, Habicht, & Seeb, 2012). This system is phenotypically diverse in terms of years spent in freshwater, with substantial expression of an “ocean‐type” life history of the Late Run, which describes juveniles that go to sea in their first year of life (Figure 2). The life history of *O. nerka* has been reviewed extensively elsewhere, but in brief, juvenile anadromous *O. nerka* rear in lakes for one to three years after emergence from the gravel, though some migrate to sea soon after emergence. After migrating to sea, adult *O. nerka* spend one to four years in the ocean before returning to freshwater, where they spawn and die in late summer and autumn (Burgner, 1991).

### 2.2 Observed smolt length of Olga lakes *Oncorhynchus nerka*

To assess smolt length at ocean entry, *O. nerka* smolts were sampled at ocean entry by the Alaska Department of Fish & Game (ADF&G) from May 20–July 30, 1990; May 11–August 4, 1991; May 5–July 31, 1992; and May 10–August 6, 1993, using a Canadian fan trap 1.6 km downstream of Lower Olga Lake (Finkle & Loewen, 2015; Figure 1). A smear of scales was pulled from individuals and mounted on glass slides; fork length was measured; and smolt scales were later used for age determination (Finkle & Loewen, 2015). Length measured directly from smolts at ocean entry will be hereafter referred to as observed smolt length.

### 2.3 Back‐calculated smolt length of returning Olga lakes *Oncorhynchus nerka*

Adult *O. nerka* have been enumerated and sampled for age and length (mid‐eye to tail fork) at the Upper Station weir since 1928. Based on stock‐specific run timing, fish returning through July 15 are considered the Early Run, where fish returning after July 15 are assumed to be the Late Run (Gomez‐Uchida et al., 2012). Run reconstructions based on escapement and scale pattern analysis are available beginning in 1969 for the Early Run and 1970 for the Late Run. As such, the collection of archived adult scales begins in 1969–1970 and continues annually by ADF&G.

Archived scales sampled by ADF&G from returning adult fish during 1969–2016 were preserved as impressions in acetate cards and include corresponding information on length, age, and sex (based on physical examination of external characteristics). Scales and impressions were included in the study based on the following criteria: (a) We agreed with ADF&G age determination, (b) annuli are clearly defined and not affected by regeneration or reabsorption of the scale, and (c) the shape of the scale indicates it was taken from the preferred area, which is immediately above the lateral line and slightly forward of the adipose fin (Koo, 1962; Ruggerone, Nielsen, & Bumgarner, 2007). Smolt scales were randomly sampled (*n* = 1,300) for each age‐class (0–3) from when smolts were sampled (1990–1993) with stratified random sampling for each age (0–2) and year (1990–1993). A random sample of age‐3 smolts was sampled across all years due to a low number of available scales (*n* = 100). Following the approach by Ruggerone, Nielsen, and Agler (2009), a random sample of 50 adult scales was selected from each returning age‐class that entered the ocean in 1990–1993; in addition, age‐classes were included in the analysis if 25 readable scales of each sex could be obtained, with a total of 50 scales per age‐class in a given year. Acetate scale impressions were digitized using a Z‐Scan 46‐II microfiche reader attached to a 19.3 mm zoom lens, and images were exported at high resolution (3,352 × 4,425 pixels) to Image‐Pro software^®^ for accurate measurements of scale patterns.

To back‐calculate smolt length from returning adult scales, we measured the distance (mm) on the longest axis from the focus of the scale to the last circulus at the end of freshwater growth. In addition, we measured the total distance from the focus of the scale to the edge of the scale for both adult and smolt scales (Ruggerone et al., 2007). Random checks between two trained readers were done to assure consistency in measurements for adult scales (~5% of samples).

**FIGURE 3:**
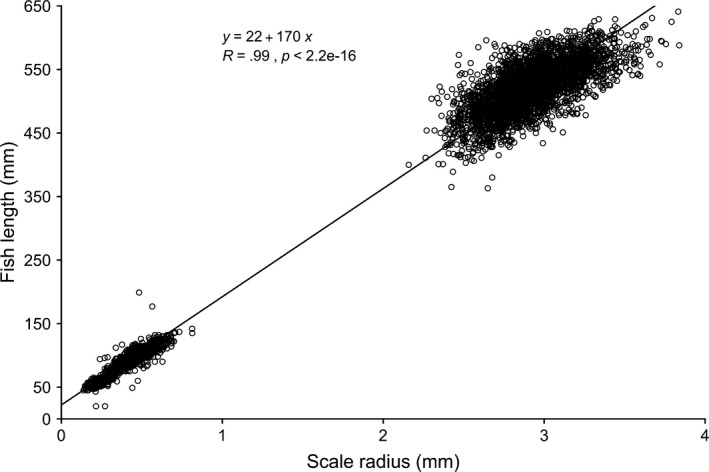
Relationship between length (mm) and scale radius (mm) based on scale and length measurements from juvenile and adult *Oncorhynchus nerka* from Olga lakes, Kodiak, AK. The intercept *c* = 22 mm, 95% CI [20.3, 23.7] and represents the theoretical length of a *O. nerka* at the beginning of scale formation

We used the Fraser–Lee equation (Fraser, 1916; Lee, 1920) to back‐calculate smolt length from scales of returning adults. The Fraser–Lee method has been widely used for many species of fish (Bond, Hayes, Hanson, & MacFarlane, 2008; Ward et al., 1989; Weitkamp, Orsi, Myers, & Francis, 2011) and is preferred because the intercept, *c*, has a biological interpretation as the length of a fish at the beginning of scale growth. In addition, the Fraser–Lee method has been verified for *O. nerka* by comparing the lengths of tagged and recaptured *O. nerka* with their scale radii (Fukuwaka & Kaeriyama, 1997). The constant *c* was obtained by calculating the regression of length on scale radius from adult and smolt scales (Figure 3):

(1) $$g\left(S\right)=c+dS,$$

where $g\left(S\right)$ is the mean body length for fish with scale radius *S, c* is the estimated intercept, and *d* is the estimated slope. The intercept represents the theoretical length of Olga lakes *O. nerka* at the time of scale formation.

The back‐calculated smolt length of returning *O. nerka* at the time of ocean entry was then calculated using the following equation:

(2) $$\text{BSL}=c+\left(L_{c}-c\right)\left(\frac{S_{f}}{S_{c}}\right),$$

where BSL is the back‐calculated smolt length, *L*_c_ is the length of the returning adult, *S*_f_ is the scale radius of the freshwater growth zone, *S*_c_ is the scale radius of the returning adult, and *c* is equivalent to *c* in Equation 1.

### 2.4 The magnitude and direction of selection on smolt size

We estimated the magnitude and direction of selection on size for Olga lakes *O. nerka* by comparing the distribution of observed smolt lengths from cohorts entering the ocean in the years 1990–1993 to the distribution of back‐calculated smolt lengths of the corresponding ocean‐entry cohort of surviving adults (following Bond et al., 2008). To demonstrate the intensity and direction of natural selection acting on body size at ocean entry during the marine phase of each ocean‐entry cohort, we calculated yearly length‐based standardized selection differentials (SSDs) for each freshwater age (Kendall, Hard, & Quinn, 2009):

(3) $${\text{SSD}}_{a_{y}}=\frac{\bar{{\text{BSL}}_{a_{y}}}-\bar{{\text{OSL}}_{a_{y}}}}{S_{{\text{OSL}}_{a_{y}}}},$$

where $\bar{{\text{OSL}}_{a_{y}}}$ is the mean observed smolt length for fish of age *a* that entered the ocean in year *y*, ${\bar{\text{BSL}}}_{a_{y}}$ is the mean back‐calculated smolt length for fish of age *a* in year *y*, and $S_{{\text{OSL}}_{a_{y}}}$ is the standard deviation of observed smolt length of age *a* in year *y*.

The yearly length‐based standardized selection differential is a measure of the difference in mean observed smolt length migrating from Olga lakes versus mean back‐calculated smolt length of surviving *O. nerka* escaping back to Olga lakes to spawn. This value was then divided by the standard deviation of observed smolt length in order to compare across years and populations (Brodie, Moore, & Janzen, 1995; Matsumura, Arlinghaus, & Dieckmann, 2012). We generated bootstrapped standard deviations for these selection differentials by performing weighted sampling of observed smolt lengths (*n* = 200) and back‐calculated smolt lengths (*n* = 200) with replacement, applying the selection formula, and then repeating this procedure 1,000 times. Observations were weighted by the relative contribution of a run (Early or Late) to the total return of an age‐class in a given year. We present the mean SSD and bootstrapped standard deviation. We explored the form of selection (e.g., disruptive and stabilizing) through visual assessment of the probability density of observed smolt length at ocean entry with the probability density of back‐calculated smolt length of the return.

### 2.5 Comparisons to global database of selection

The relative magnitude of selection was determined by comparing SSDs to a database of linear selection differentials compiled by Kingsolver et al. (2001) and updated by Siepielski et al. (2017). The database includes 3,759 estimates from 91 different species, including both terrestrial and aquatic taxa. Selection differentials included in the database were replicated either spatially or temporally and use survival or fecundity to measure fitness. The majority of traits considered were morphological, though life history, behavior, and phenology traits were included as well (see Siepielski et al., 2017). We calculated percentiles of the global database selection differentials based on comparisons against the entire database (all categories of traits) and separately to a subset of the database comprised of just morphological traits, such as body size. If a selection differential fell outside of the 90th percentile, selection was considered strong relative to estimates in the database.

### 2.6 Relationship of magnitude of selection and ocean‐entry timing

We explored the association between the magnitude of selection with mean ocean‐entry timing by fitting a linear model that was specified as follows:

(4) $${\left|\text{SSD}\right|}_{a_{y}}=\alpha +*{\bar{\text{Timing}}}_{a_{y}}+\varepsilon_{a_{y}}\;\varepsilon_{a_{y}}∼N\left(0,\sigma^{2}\right),$$

where ${\left|\text{SSD}\right|}_{a_{y}}$ is the magnitude of selection on size of age *a* smolts in year *y* and ${\bar{\text{Timing}}}_{a_{y}}$ is the mean ocean‐entry timing (DOY) of smolts of age *a* in year *y,* and $\varepsilon_{a_{y}}$ is the observed error. The effect of timing on the magnitude of selection was then quantified using the parameter estimates from the model and confidence intervals were calculated.

## 3 RESULTS

### 3.1 Observed smolt length of Olga lakes *Oncorhynchus nerka*

A total of 14,401 smolts were sampled by ADF&G from 1990 to 1993, which include juveniles of age 0 (*n* = 6,653), age 1 (*n* = 2,572), age 2 (*n* = 5,176), and age 3 (*n* = 181). The mean observed length of age‐0, age‐1, age‐2, and age‐3 smolts was 58.5 mm, 91.4 mm, 103.3 mm, and 113.4 mm, respectively, and the mean length of each age‐class varied significantly across years (ANOVA, *F*_2_ = 216.09, *p* < .001; Table 1). Freshwater age‐3 fish were excluded from further analyses due to a small number of freshwater age‐3 fish at ocean entry.

**TABLE 1:** Summary of observed smolt length (OSL), back‐calculated smolt length (BSL) of returning adults, selection on size, and ocean‐entry timing of age‐0, age‐1, and age‐2 smolts of Olga lakes. Standardized selection differentials (SSD) are sd‐standardized. Standard deviation is shown in parentheses

Age	Year	Sample size (OSL/BSL)	Mean OSL (mm)	Mean BSL (mm)	Difference in mean length (mm)	SSD	Mean day of ocean entry
0	1990	939/100	54.5 (5.9)	66.9 (6.3)	22.8	2.1 (0.2)	July 17
	1991	1622/100	59.3 (6.8)	79.0 (10.7)	34.8	3.3 (0.3)	July 21
	1992	1783/100	57.5 (3.7)	76.8 (12.1)	33.5	5.2 (0.4)	July 17
	1993	2309/50	60.5 (5.7)	73.0 (11.5)	20.7	2.2 (0.2)	July 15
1	1990	325/50	81.4 (7.3)	66.6 (7.8)	−18.2	−2.0 (0.1)	June 15
	1991	658/100	93.7 (7.6)	87.2 (17.9)	−6.9	−2.4 (0.3)	June 17
	1992	476/100	93.5 (7.3)	94.8 (15.2)	1.4	−0.5 (0.2)	June 17
	1993	1113/200	92.0 (5.8)	86.0 (15.3)	−6.6	−1.0 (0.2)	June 7
2	1990	1539/150	99.7 (7.5)	119.0 (20.5)	19.2	2.6 (0.2)	June 10
	1991	947/100	102.3 (9.4)	118.4 (9.6)	15.7	1.7 (0.1)	June 9
	1992	1837/100	103.1 (7.2)	118.0 (9.3)	14.5	1.8 (0.1)	June 6
	1993	853/100	111.4 (12.0)	125.3 (11.0)	12.5	1.0 (0.1)	May 26

### 3.2 Back‐calculated smolt length of returning Olga lakes *Oncorhynchus nerka*

**FIGURE 4:**
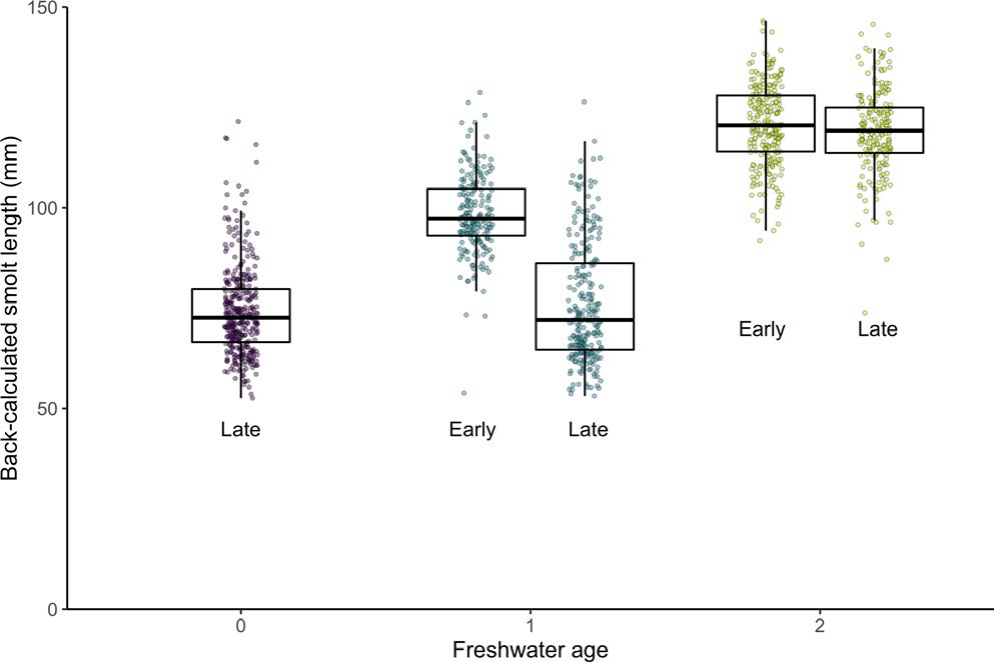
Mean back‐calculated smolt length of Olga lakes returning adults among three freshwater ages of ocean‐entry years 1990–1993. Points indicate individual observations of smolt length pooled across years. Both the Early Run (return from May to mid‐July) and Late Run (return from mid‐July to September) are shown here, where freshwater age‐0 fish are only present in the Late Run

A total of 1,250 adult *O. nerka* scales from the Olga lakes return (1992–1996) were digitized and measured, which includes fish of freshwater age 0 (*n* = 350), age 1 (*n* = 450), and age 2 (*n* = 450). The intercept *c* of the linear regression of fish length on scale radius from smolt (*n* = 1,300) and adult (*n* = 2,983) scales was estimated as 22 mm. The average back‐calculated smolt length of freshwater age‐0, age‐1, and age‐2 returning adults was 74.3 mm, 86.0 mm, and 120.0 mm, respectively, and varied significantly across ocean‐entry years (ANOVA, *F*_2_ = 20.532, *p* < .001; Table 1). Mean back‐calculated smolt length differed significantly between the Early and Late Run only for age‐1 smolts (*t* test, *t* = 19.09, *p* < .001), where Early Run fish were, on average, 19% larger (Figure 4). Freshwater age‐0 fish comprise <5% of the Early Run return; therefore, there was an insufficient number of scales to include this age‐class from the Early Run in our analyses. In addition, Early Run freshwater age‐1 fish from ocean‐entry year 1990 were not included due to an insufficient number of readable scales available.

### 3.3 The magnitude and direction of selection on smolt size

**FIGURE 5:**
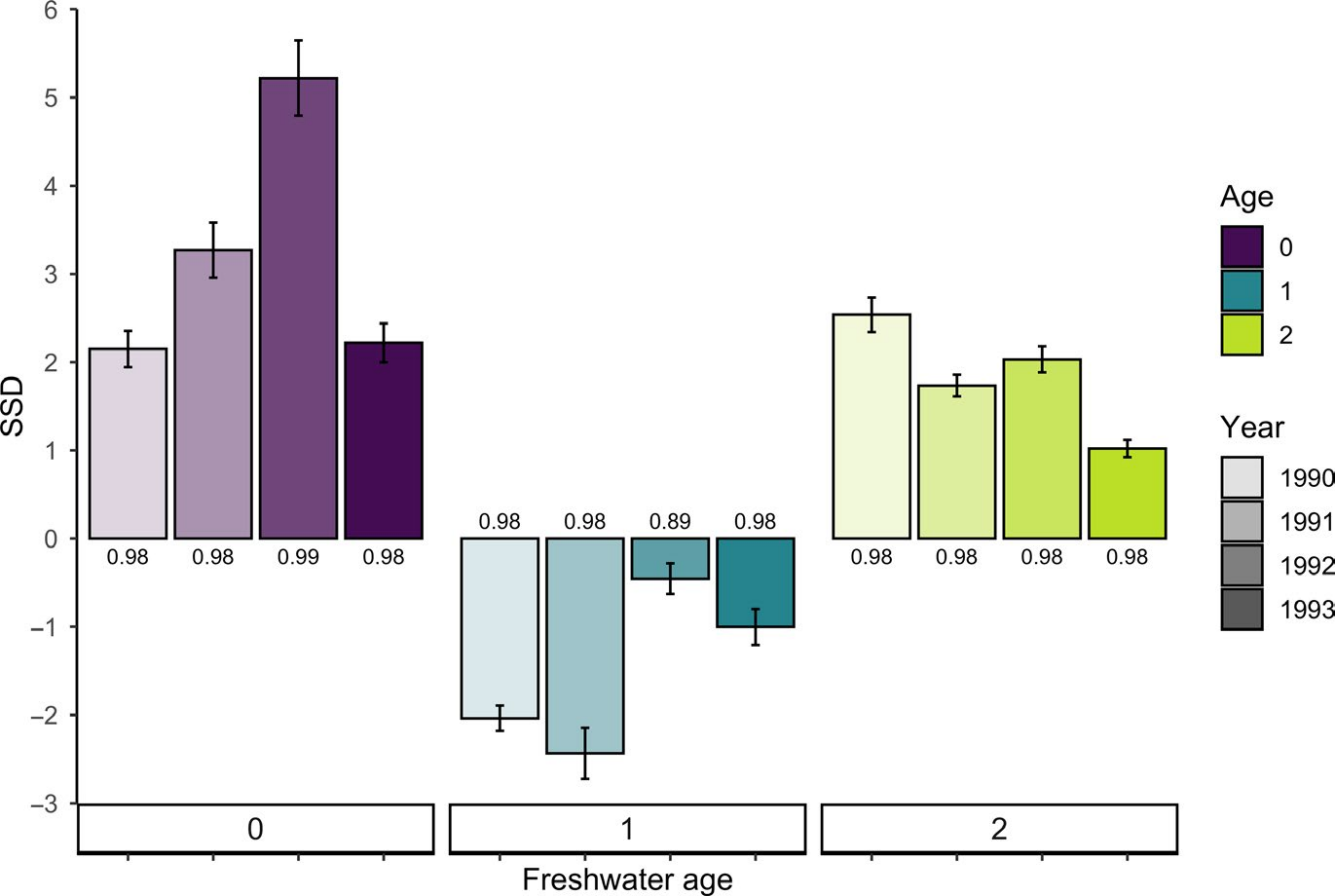
Standardized selection differentials (SSDs) measuring the magnitude of selection for smolts, where a positive SSD indicates that selection favored larger individuals and a negative SSD indicates that selection favored smaller individuals. Graph depicts SSDs of three different freshwater age‐classes across ocean‐entry years of 1990–1993. Error bars indicate standard deviation of bootstrapped (*r* = 1,000) mean, and labels indicate comparison to a global database of linear SSDs (Siepielski et al., 2017). For example, a value of 0.98 indicates relatively strong selection as the SSD falls outside of the 98th percentile compared to 3,759 different estimates of quantified selection on traits of 91 different species

Standardized selection differentials (SSDs) ranged from −2.42 (selection favored smaller individuals) to 5.19 (selection favored larger individuals) indicating that the average size of age‐classes after selection differed by 2.42 to 5.19 standard deviations than the average size before selection. SSDs varied significantly among age‐classes (ANOVA, *F*_2_ = 26.87, *p* < .001); however, SSDs did not vary significantly across years (ANOVA, *F*_2_ = 1.78, *p* = .25; Figure 5). Selection for larger smolts was observed for age‐0 and age‐2 smolts in all years, with a mean SSD of 3.21 (*s* = 1.44) and 1.83 (*s* = 0.63), respectively, for ocean‐entry years 1990–1993. In contrast, for age‐1 fish, selection favored smaller smolts and we observed a mean SSD of −1.49 (*s* = 0.91).

**FIGURE 6:**
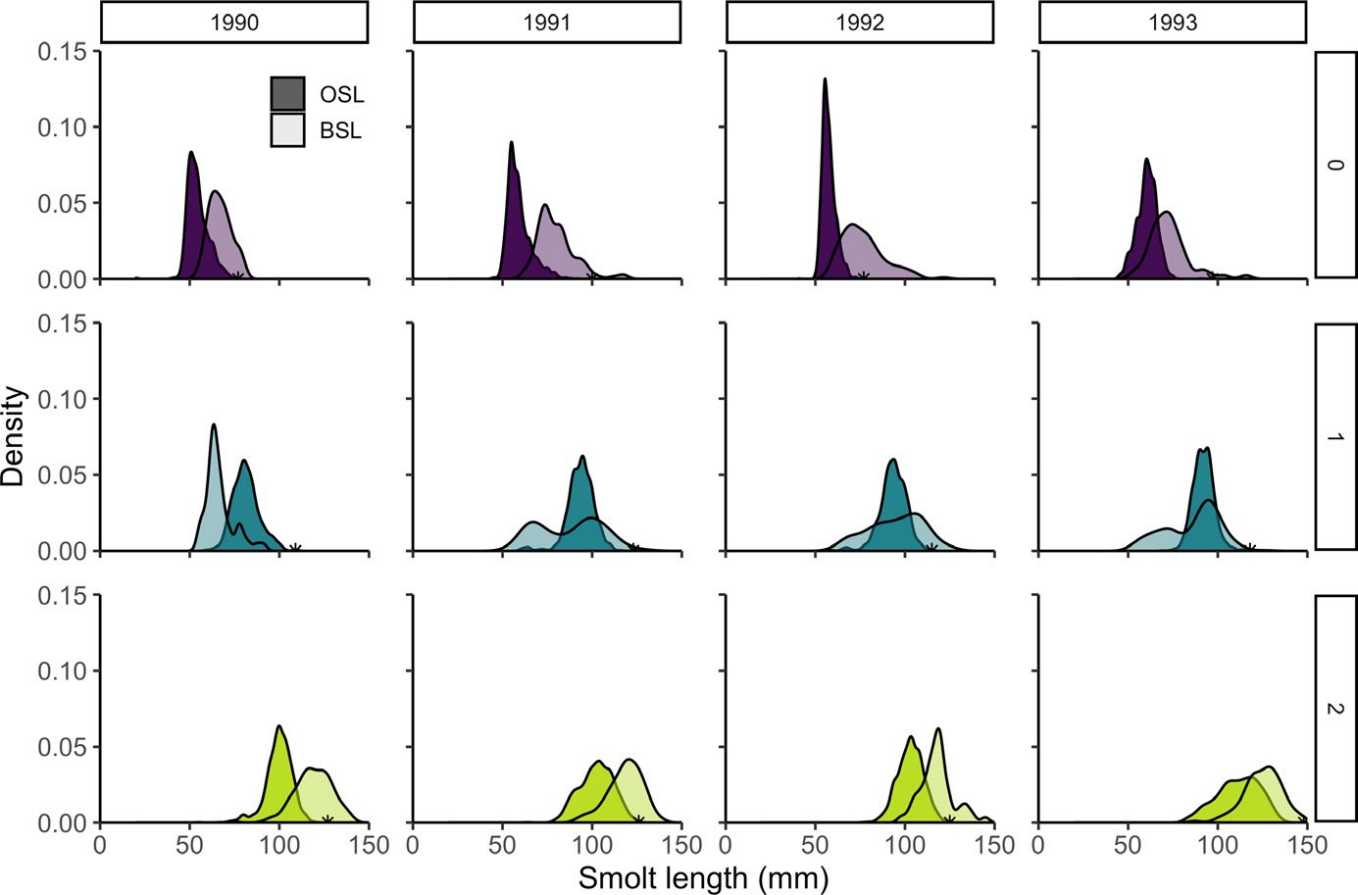
Probability density of observed smolt length (OSL) at ocean entry (dark) and back‐calculated smolt length (BSL) of returning adults (light) for three different age‐classes from years 1990–1993 of ocean entry. Purple, green, and orange colors correspond to freshwater age 0, age 1, and age 2, respectively. Global difference in mean length of OSL and BSL was significant (ANOVA, *F*_1_ = 214.02, *p* < .001). Star indicates the maximum observed smolt length for a given year and age

Probability density functions comparing observed smolt length at ocean entry and back‐calculated smolt length of returns show changes in the distribution shape after selection for some marine cohorts. We detected evidence of bimodal distributions of length after selection for age‐1 cohorts in 1991 and 1993 (Figure 6), consistent with disruptive selection. For age‐0 cohorts, length distributions had higher variance after selection, but consistently displayed directional selection with a shift toward larger size. In contrast, age‐2 length distributions appeared to maintain a similar distribution while shifting to a larger mean body size after selection (Figure 6).

### 3.4 Comparisons to global database of selection

The estimates of selection on Olga lakes smolt body size were strong compared to the global database of standardized selection differentials comprised of traits including morphology, physiology, behavior, and life history. We note that comparisons were similar when selection differentials of only morphological traits or body size were retained, and therefore, we have only reported the analysis comparing selection differentials of all traits in the global database. Standardized linear selection differentials from Siepielski et al. (2017) had a mean magnitude of 0.18 (*s = *0.32), whereas the selection on size for Olga lakes *O. nerka* had a mean magnitude of 2.18 (*s = *1.23). All twelve of the estimated SSDs fell outside of the 90th percentile of global selection estimates, with the SSD corresponding to age‐0 smolts in 1992 falling outside of the 99th percentile (Figure 5).

### 3.5 Relationship of magnitude of selection and ocean‐entry timing

**FIGURE 7:**
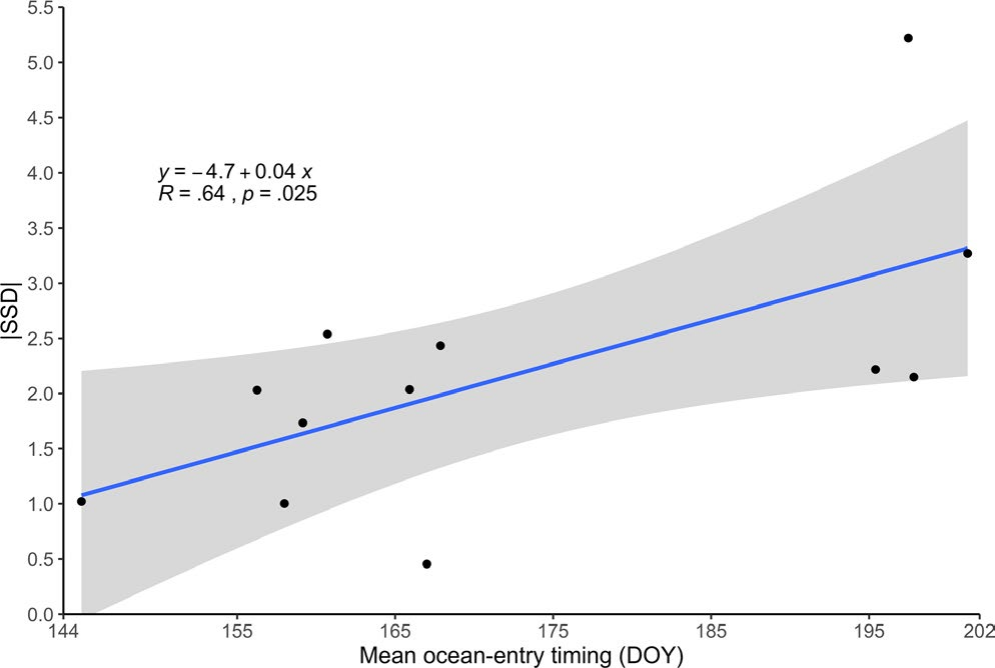
Linear regression of the magnitude of selection on smolt size (|SSD|) with ocean‐entry timing (DOY), where the shaded area indicates 95% confidence intervals. The parameter estimate of ocean‐entry timing (DOY) was 0.04, 95% CI [0.006, 0.07] with an intercept of −4.7, 95% CI [−10.5, 1.2]

The mean day of ocean entry for Olga lakes *O. nerka* during the years 1990–1993 was approximately June 26th (*s* = 3.5 days) and varied significantly among age‐classes (ANOVA, *F*_2_ = 242.16, *p* < .001) and years (ANOVA, *F*_3_ = 7.13, *p* = .02). Older smolts tended to migrate to the ocean at an earlier date than younger age‐classes (Figure S1), consistent with patterns generally observed in Pacific salmon. On average and across years, age‐2 smolts entered the ocean at the earliest date (June 5th, *s* = 7 days), followed by age‐1 smolts (June 14th, *s* = 4.5 days), and the mean ocean‐entry date of age‐0 smolts was 33 days later in the season (July 17th, *s* = 2.4 days). The absolute magnitude of selection was positively correlated to late ocean‐entry timing where the magnitude of selection increased by 0.04, 95% CI [0.006, 0.07], with each mean DOY that an age‐class migrates later in the season (*R* = 0.65, *p* = .025; Figure 7).

## 4 DISCUSSION

Our findings indicate that selection on body size of migrating anadromous juvenile salmon can vary in magnitude and direction among freshwater age‐classes within a watershed, but trends can also be generally consistent among years. Furthermore, the magnitude of selection on size was strong relative to linear selection values reported for other natural populations. The magnitude of selection appeared to be positively correlated with late mean ocean‐entry timing. Taken together, our results demonstrate that bigger is not always better regarding *O. nerka* smolt survival and smolts can experience relatively strong positive or negative selection on size after they enter the ocean, depending on the life history “decision” of what year to migrate to sea.

### 4.1 The magnitude and direction of selection on smolt size

The present study is the first to quantify the magnitude and direction of selection on size in a Pacific salmon system that includes variable age‐classes while also comparing selection experienced by cohorts across years. We hypothesized that selection would favor large individuals, to some extent, within all freshwater age‐classes (age 0–age 2). In line with our hypothesis, our data reveal consistently strong positive selection for both age‐0 and age‐2 smolts; however, unexpectedly, we observed negative selection on size for age‐1 smolts. Overall, our findings corroborate the general understanding that smolts of a larger size may have a survival advantage over their smaller counterparts (Foerster, 1954; Henderson & Cass, 1991; Koenings & Geiger, 1993). Differential survival of large individuals could be attributed to size‐biased consumption by a predator as a result of gape limitations, behavioral selection by a predator, or increased escape ability with smolt size (Sogard, 1997). The aggregation of predators (e.g., *Mergus* spp.*,* mergansers; *Larus* and *Chroicocephelus* spp.*,* gulls; and *Lontra canadensis,* river otters) at lake outlets during out‐migration of salmon smolts has been documented in other systems (Clark & Furey, 2016). Other known fish predators of Pacific salmon smolts include *Squalus acanthias* (spiny dog fish), *Lampetra ayresi* (river lamprey), *Leptocottus armatus* (Pacific staghorn sculpin), and *Anoplopoma fimbria* (sablefish) though this is not an exhaustive list (Beamish & Neville, 1995; Beamish, Thomson, & McFarlane, 1992; Sturdevant, Sigler, & Orsi, 2009; Whitney, Beaudreau, & Duncan, 2017). Salmon smolts are also predated on by seabirds throughout their range (see Cederholm et al., 2000); predation by *Cerorhinca monocerata* (rhinoceros auklets) has been shown to be size‐selective, where prey items were consistently shorter and lighter for their length than the general population (Tucker, Hipfner, & Trudel, 2016).

Contrary to our hypothesis, we observed negative selection on size for age‐1 *O. nerka* smolts. There is limited evidence for the selection against large juvenile salmonids, though Carlson, Hendry, and Letcher (2004) found that selection sometimes favored small/fast‐growing *Salmo trutta* (wild trout). In some cases, bird predation can be biased by preferentially selecting large or intermediate sized individuals (Sogard, 1997). *O. mykiss* (steelhead trout) smolts of intermediate length (145–190 mm) were more likely to be eaten by gulls, whereas smolts below or above this range were much less vulnerable to predation (Osterback et al., 2014). In pond experiments investigating size‐selective predation on two size‐classes of *Leiostomus xanthurus* (spot), small individuals were more likely to survive when the predator field was composed of large predators (Rice, Crowder, & Rose, 1993). Harbor seals have recently been documented to preferentially consume large‐bodied juvenile salmon (i.e., *O. kisutch*, *O. tshawytscha*, and *O. nerka*) in the Strait of Georgia (Thomas, Nelson, Lance, Deagle, & Trites, 2017), and therefore, large age‐1 smolts could be more vulnerable to this type of predation. Overall, it appears that predation can be biased toward either large or small individuals; therefore, size‐selective survival will likely be a result of the relative levels of mortality by each predator group (Nelson et al., 2019). If smolt age‐classes are separated in space (i.e., habitat use) or time (i.e., out‐migration timing) and encounter different predator landscapes, hypothetically, this could result in differences in selection on size. However, one would expect this to be dynamic among years and this hypothesis may not reflect the consistent patterns of selection we have observed here.

It is difficult to determine whether negative selection was a function of the mean size of age‐1 smolts, ocean‐entry timing, or an interaction of the two, as both are intrinsically correlated (Quinn et al., 2009). Here, we observed that small, presumably fast‐growing, age‐1 smolts entered the ocean earlier, but at a smaller size than their slower growing counterparts that entered later, at a larger size. The timing of ocean entry is an important life‐history trait that can strongly influence the early marine survival of salmon smolts (Quinn, 2018). Columbia River basin *O. tshawytscha* and *O. mykiss* juveniles that migrated from early to mid‐May had up to a 50‐fold higher survival rate than those migrating in mid‐June (Scheuerell et al., 2009). For hatchery‐origin fall run *O. tshawytscha* in California, release time relative to spring transition, among other factors, was a useful predictor of ocean survival rates (Satterthwaite et al., 2014). In hatchery operations, where size and release date can be manipulated, it has been shown that for three size‐classes released on four different dates, date of release had a strong effect on survival, whereas there was little variation in survival among size‐classes within dates (Bilton et al., 1982; Quinn, 2018). Similarly, in several Alaska lakes, age‐2 smolts had lower smolt‐to‐adult survival than comparably sized age‐1 smolts until both reached approximately 100 mm (Koenings & Geiger, 1993). The authors hypothesized that smaller age‐1 smolts that migrated early could incur a survival advantage as predators may be satiated by large age‐2 smolts migrating at the same time. This was demonstrated in the Kvichak system in Bristol Bay, Alaska where percentages of age‐2 smolts were significantly and positively correlated with smolt‐to‐adult survival of co‐migrating age‐1 smolts (Koenings & Geiger, 1993; Tillotson & Quinn, 2016). If selection favors smolts that migrate earlier in the season, this would explain why we observed positive selection for age‐0 and age‐2 smolts, as they decreased in size‐at‐age throughout the season, whereas age‐1 smolts increased in size‐at‐age.

It is likely that there is not a single combination of size and ocean‐entry timing that maximizes survival, but several optima may occur as a result of the interaction of the effect of size and timing. Our results support this hypothesis, although selection for age‐1 smolt size was negative overall, back‐calculated smolt length after selection was commonly bimodal, suggestive of disruptive selection (Brodie et al., 1995). However, there appears to be evidence of stock effects, where negative selection was driven mainly by higher survival of small individuals of the more abundant Late Run stock, whereas survivors of the Early Run stock were closer to the mean observed smolt length at ocean entry. Furthermore, even though we cannot assign out‐migrating smolts to a specific stock, there does not appear to be any evidence of a bimodal distribution of age‐1 smolt length at ocean entry; therefore, dramatic differences between stocks seem to appear after selection. One hypothesis is that due to earlier spawning by adults, Early Run juveniles hatch earlier and are therefore larger, on average, than Late Run smolts out‐migrating at the same time (Sparks et al., 2019). Carried forward, if Early Run age‐1 smolts migrate disproportionately early and at a larger size, than selection on early migration timing may not result in negative selection on size as observed for Late Run smolts. Ultimately, we do not know the mechanisms causing the observed patterns of selection on size but have demonstrated the complexity of the relationship between smolt size and survival by quantifying selection on size within multiple age‐classes.

### 4.2 Comparisons to global database of selection

Despite variation in selection estimates among age‐classes, selection was strong compared to estimates of selection in other natural populations. For example, linear selection differentials for the 3,759 estimates of 91 species compiled by Siepielski et al. (2017) have a mean absolute value of 0.18, where the mean absolute value in the present study was 2.14. All selection differentials for size were outside the 90th percentile, which suggests that smolts experience strong size‐selective pressure, either positive or negative, after they enter the ocean. We acknowledge several limitations and caveats of the present study design regarding quantifying the strength of selection on size that may underpin the large estimates of selection observed. First, the back‐calculation method used to estimate smolt size distributions from returning adults may have introduced bias that is interpreted as strong selection. Indeed, the methodology used can introduce inflated variance (Wilson, Vigliola, & Meekan, 2009) and is sensitive to sampling effort (Beacham, Araujo, Tucker, & Trudel, 2018; Siepielski et al., 2009). Second, sampling bias of migrating smolts or returning adults may contribute to our estimates. For three of the years studied (1990–1992), approximately 13%–22% of back‐calculated lengths were larger than the maximum observed length at ocean entry. Since this was only an issue for age‐2 smolts, it suggests that the largest, oldest smolts were under‐represented in the sampled juvenile population during seaward migration and is consistent with a mismatch between timing of sampling and migration of the largest age‐2 smolts or size‐dependent gear avoidance. Finkle and Harding (2015) observed that large smolts were efficient at avoiding the Canadian fan trap by swimming into then out of the trap. Therefore, it is highly probable that selection estimates for age‐2 smolts are biased high. Third, smolt length was back‐calculated from scales of adults from the escapement after late‐stage predation and fishing mortality has occurred that could potentially introduce bias in selection estimates (Hanson et al., 2010). Previous work has found that Pacific salmon have a higher probability of maturing at an earlier age (i.e., smaller at return) if they were large as smolts (Bilton, 1971; Vøllestad, Peterson, & Quinn, 2004). Olga lakes *O. nerka* are subject to both a purse seine and gillnet fishery; purse seine fisheries can disproportionately catch larger males and smaller females, whereas gillnet fisheries generally catch larger fish depending on mesh size (Kendall et al., 2009; Kendall & Quinn, 2012). Therefore, the distribution of back‐calculated smolt length could be biased high as a disproportionate number of small adults escaped into the system that are predicted to have been larger at ocean entry. Finally, our analyses assume direct selection on body size, rather than indirect selection operating through other correlated traits. We acknowledge that body size and seaward migration timing, both which are thought to influence survival in salmonids, are correlated. For example, age‐2 smolts that leave early may have both the advantage of entering the ocean at an optimal time, as well as having a larger body size (Figure S1). Thus, the magnitude of selection on “size” could be a compounded effect of selection on the phenotypic traits of body size and run timing.

Despite these caveats, we are confident that our selection estimates are not solely a result of biased methodology, juvenile sampling, or selection acting indirectly on correlated traits. First, the scale radius of out‐migrating smolts was directly comparable to the measure of freshwater growth on adult scales and reflected patterns of back‐calculated lengths (Figure S2) and parallel analyses with other back‐calculation methods (e.g., linear log–log regression and weighted regression) produced similar trends in selection on size. Second, it seems improbable that smaller age‐2 and age‐0 juveniles would have a higher probability of capture in the sampling gear, while smaller age‐1 juveniles would have a lower probability of capture. Though we do acknowledge that the largest age‐2 juveniles may be under‐represented, there does not appear to be a systematic bias across all age‐classes. We note the assessment of Hendry (2016) that compared to other traits, body size suffers from a positive bias in the estimation of selection. Third, back‐calculated smolt length was only significantly different by saltwater age for freshwater age‐0 fish; even then, saltwater age‐3 fish tended to be larger as smolts. Furthermore, the strength of correlation between smolt length and adult length was not significantly associated with the magnitude of selection on size. Finally, we acknowledge that selection rarely acts on individual traits directly, but rather through indirect selection on correlated traits. Because we do not know the smolt migration timing of returning adults, we are unable to separate direct from indirect selection. However, evidence provided by Kingsolver and Diamond (2011) indicates that estimates of selection differentials (i.e., direct selection on individual traits) are strongly correlated with selection gradients (i.e., indirect selection on individual traits) across studies and taxa. This suggests that our interpretation of strong selection may not have changed even if quantification of indirect selection had been possible.

Alternatively, the strong estimated selection on size may be the result of an increase in selection opportunity due to a survival bottleneck in which very few smolts survive to be mature adults. For example, across multiple wild or naturally rearing populations of *O. nerka* populations, the average smolt‐to‐adult survival was only 13.1% (Quinn, 2018). Further, the strongest values of selection on size that we observed were for age‐0 smolts, which have the lowest estimated marine survival, often lower than 1% (Quinn, 2018). More generally, the early life history of fishes is commonly associated with periods of high mortality, increasing the opportunity for agents of selection to drastically change the distribution of traits within a population (Conover & Schultz, 1997). The global database of selection differentials is mainly comprised of terrestrial taxa since estimates of selection that are replicated in time or space for aquatic taxa are much less common (Siepielski et al., 2017). Therefore, the levels of mortality that stable, terrestrial populations experience may be magnitudes lower than the rates of survival that are likely operating here. Though, we recognize that truncation selection rarely occurs in nature as other traits are likely under selection and there is usually no threshold phenotypic value that determines reproductive success or survival (Matsumura et al., 2012). Thus, it is most prudent to interpret the magnitudes of selection to be relative among age‐classes within our study system and cautiously compared to other studies. Our analysis, like all analyses of selection, is sensitive to sampling design and potential biases. Yet despite these potential biases, we interpret the magnitude of selection revealed here to be substantial.

With such strong estimated selection on size, we might expect that there would be a mean shift in the phenotype of this population over time, given that body size (heritability, *h* ^2^ ~ 0.44) and timing of ocean entry (*h* ^2^ = 0.23) are at least in part genetically controlled (Carlson & Seamons, 2008). Though the present study does not aim to predict the evolution of body size in *O. nerka* smolts, there are reasons to consider why selection on size may not result in an evolutionary response. First, by quantifying size‐selective survival from smolt to adult we ultimately ignore the trade‐offs between growth potential and predation risk (Sogard, 1997). Growth may be limited by physiological constraints or competing requirements such as immune capacity or response to environmental stress (Arendt, 2018; Conover & Schultz, 1997). Although large smolts may survive at higher rates than smaller smolts, large body size comes at the cost of additional time and risk of predation for juvenile fish in freshwater before they make it to the ocean (Quinn et al., 2009). Therefore, our estimates measure the strength of selection acting on smolt size during a portion of the life history and do not represent cumulative selection acting on smolt size (Matsumura et al., 2012). Second, ocean conditions may be variable from year to year and within a season, reflecting a shifting optimum, which in turn would be expected to maintain the expression of variable age and size structure within a population. For instance, an individual would benefit from producing offspring of variable ages and size to “hedge its bets” and increase the probability that one cohort will experience favorable conditions (Carr‐Harris et al., 2018; Schroeder et al., 2016).

### 4.3 Relationship of magnitude of selection and ocean‐entry timing

By investigating mean ocean‐entry timing, we were able to demonstrate an association with the magnitude of selection on smolt size. Cohorts that migrated earlier in the season tended to experience weaker selection on size; however, this relationship was mainly driven by differences in freshwater age. It is extremely challenging to identify the mechanisms that underpin size‐selective survival for salmon as they enter the ocean, and therefore, the ecological agents of selection are largely undescribed in the literature (Duncan & Beaudreau, 2019). Though the present study was not designed to identify the mechanisms behind size‐selective survival, several agents of selection may contribute to the observed variation in size‐selective survival. For example, year‐to‐year variation in parasitism rates may affect the magnitude of selection on size as infected juveniles that survive to the smolt stage exhibit reduced seawater adaptation, growth, and survival (Boyce, 1979; Boyce & Clarke, 1983). Environmental factors such as the strength of spring upwelling and greater freshwater discharge can contribute to higher productivity and possibly less predation risk (Kohan, Mueter, Orsi, & McPhee, 2017; Scheuerell & Williams, 2005). If marine conditions, such as these, are less favorable, opportunities for compensatory growth may be limited, increasing the disparity among size‐classes and the opportunity for selection (Nicieza & Brana, 1993). Smolt densities may also affect the magnitude of size‐selective mortality via predators; mortality rate has been shown to decrease as overall smolt abundance increases, sometimes resulting in negligible prey size‐selection effects (Furey et al., 2016; Wood, 1987). It is more probable, however, that a combination of these or other factors are responsible for the observed trends in selection on size for Olga lakes *O. nerka*.

## 5 CONCLUSIONS

In general, population data indicate that smolts of larger average size tend to have higher marine survival (Ricker, 1962) and this might lead to the assumption of the bigger‐is‐better hypothesis within smolt age‐classes as well. Here, we demonstrate that, within an age‐class, survival can favor large or small individuals depending on freshwater age. The confounded nature of migration timing and body size makes interpretation difficult and experimental approaches that isolate the effects of run timing, age, and size, and the investigation of specific size‐selective mechanisms would greatly increase our understanding of early marine survival of juvenile salmon with complex life histories.

Size‐dependent survival has long been a concern for both conservation efforts and hatchery operations (Bilton et al., 1982; Zabel & Williams, 2009) and will only continue to be as opportunities for freshwater growth are subject to change due to shifts in climate and anthropogenic disturbance or mitigation (Finstad, Einum, Forseth, & Ugedal, 2007; Hyatt, McQueen, Shortreed, & Rankin, 2004; Schindler, Rogers, Scheuerell, & Abrey, 2005). Overall, we build on previous work that describes the importance of diverse juvenile life histories for a population's resiliency to inter‐annual variation (Carr‐Harris et al., 2018; Schroeder et al., 2016). In addition, it underscores the maintenance of the processes that yield life‐history variation in Pacific salmon (Schindler et al., 2010). As lake and stream temperatures increase, decreasing thermal heterogeneity and homogenizing growth opportunities, habitat complexity may become critical for salmon populations. Therefore, the dynamics of freshwater growth and size‐selective survival should be considered especially relevant when predicting future outcomes for pristine and threatened salmon populations.

## CONFLICT OF INTEREST

None declared.

## Supporting information

Fig S1

Fig S2

## Data Availability

Data for this study are available at Dryad database https://doi.org/10.5061/dryad.qnk98sfch
