# Supplementary figures and images for: Direction and magnitude of natural selection on body size differ among age‐classes of seaward‐migrating Pacific salmon
Source: Evol Appl. 2020 Apr 9;13(8):2000–13. doi: 10.1111/eva.12957 (PMC7463379; doi:10.1111/eva.12957)

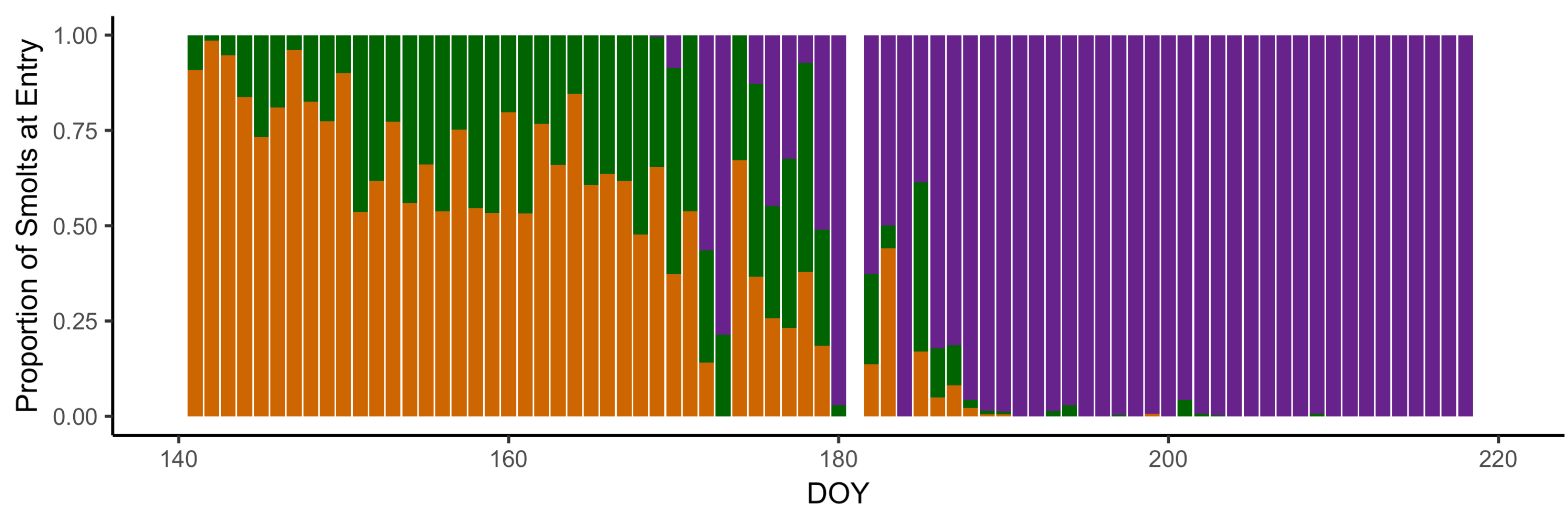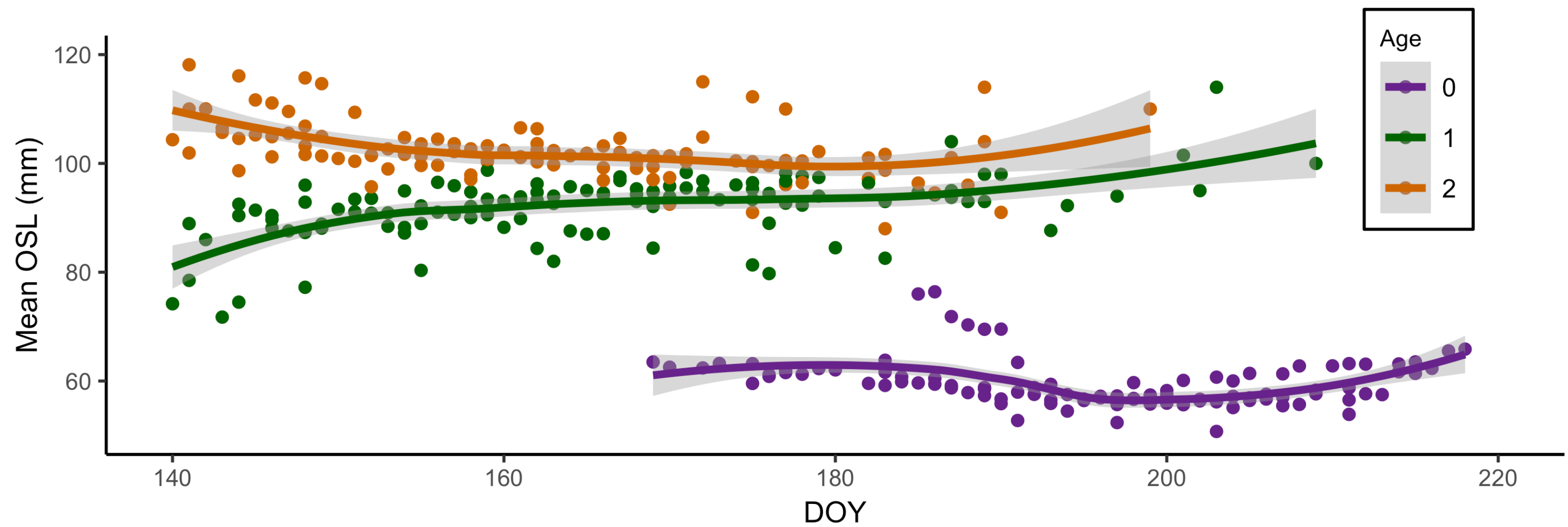

Supplement: Supplementary file 1 — Fig S1 [file EVA-13-2000-s001.pdf]

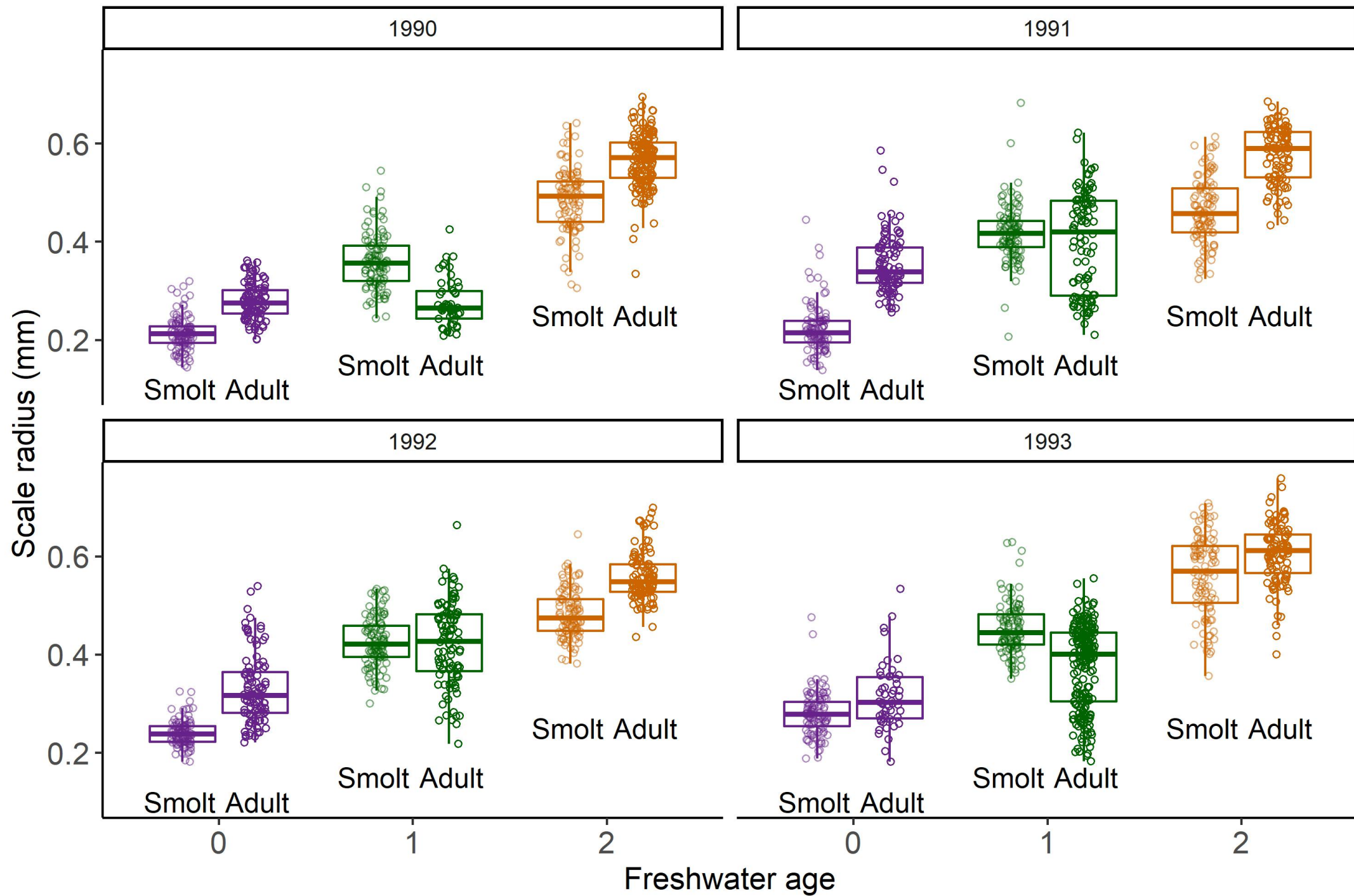

Supplement: Supplementary file 2 — Fig S2 [file EVA-13-2000-s002.pdf]
